# Collectanea Medica: Consisting of Anecdotes, Facts, Extracts, Illustrations, &c.

**Published:** 1825-07

**Authors:** 


### COLLECTANEA MEDICA:

CONSISTING OF

### ANECDOTES, FACTS, EXTRACTS, ILLUSTRATIONS, &c.

*Relating to the History or the Art of Medicine, and the Collateral Sciences.*

Floriferus, ut apes, in saltibus omnia libant,

Omnia nos, itidem, depascimur aurea dicta.

### ART. I.—*Extracts from* MR. SHAW's "*Further Observations on the Lateral or Serpentine Curvature of the Spine.*"

#### 1. *On the Effects of artificial Supports, when applied for the purpose of preventing or curing Distortions of the Spine.*

THE attempt to cure distortions by machinery fixed to the body, is a very important subject, and has been fully entered into in the preceding volumes. I shall here offer a few remarks on the application of stays, or other artificial supports. The various opinions regarding their effects show the difficulty of deciding whether or no such applications are advisable. The general impression is, that stays are hurtful: some persons, however, insist that they are useful and necessary; perhaps swayed by observing how much more common deformity is now, than when it was the custom to put children into stiff French stays almost as soon as they were born.

This question will not appear simplified by asserting that stiff stays may produce distortion, and yet that those which are generally worn are not stiff enough. This opinion suggests an explanation, which will, perhaps, put the question in a new point of view.

It is not surprising that men acquainted with the natural form of the chest, should object to children being put into such a state of bondage as they were, when stays similar to those represented in the sketch were worn.* All anatomists, who have touched upon the subject, have agreed that the effect of the support and restraint caused by such stays, is to weaken the muscles by which the spinal column, and the parts attached to it, are naturally sustained. They have also expressed their opinion that, when such a support has been used for some time, it becomes absolutely necessary.

We have almost daily opportunities of proving the correctness of the opinion, that, if any part of the body be not used, it wastes and loses its natural character. Now, as the spine cannot of itself support its own weight, and still less that of the upper part of the body, it must have the assistance of the several muscles; and as these, to be of any avail, must be kept constantly in play, it follows that, if stays be worn of such a form and substance as to prevent the action of these muscles in

* The sketch represents a figure clad in long, and apparently very stiff, stays.

supporting the spine, the muscles must waste, and become nearly useless. (See the chapter on *appropriate Exercises.)*

This appears so obvious as scarcely to require the support of names; but, to impress it more strongly, I shall quote the opinions given by some of those writers who are deservedly considered authorities in every question relating to medical science.

The following remarks are made by Portal, a very eminent French physician, on the strong and stiff stays in fashion at the time when he wrote, which was soon after the publication of the work from which the sketch in the preceding page was taken:—" Those who use them are sure to become distorted, for the muscles of the spine have been so weakened by want of use, that, when the artificial props are removed, they are no longer capable of supporting the body."

Van Swieten, the Dutch physician, whose name is illustrious in the annals of medicine, gives even a more distressing picture of the condition into which women may fall, who have been accustomed from their infancy to wear stiff stays. But the name *lorica* (coat of mail), by which he designates them, and his observations, would lead us to believe that the stays worn in his day were peculiarly stiff and strong.—" Those who have been long accustomed to wear *loricæ* can never lay them aside, for fear of the chest falling forwards, in consequence of the weakened state of those muscles which, when properly exercised, are not only capable of supporting the weight of the upper part of the body, but even of heavy burdens. Indeed, I could not view but with pity those who were so wretchedly reduced as not to dare to take off the stays even to go to sleep, much less to raise themselves, or to keep the body erect, if brought into that position."

We can conceive the bad effects that must have ensued from wearing such machines: indeed, the consequences are well described by an eminent author, who wrote about sixty years ago. His observations are so just and so applicable to the present question, that they are worthy of being quoted:—" *Some nations have fancied that nature did not give a good shape to the head, and thought it would be better to mould it into the form of a sugar-loaf. The Chinese think a woman's foot much handsomer, if squeezed into a third part of its natural size. Some African nations have a like quarrel with the shape of the nose, which they think ought to be laid as flat as possible with the face. We laugh at the folly, and are shocked at the cruelty of these barbarians; but think it a very clear case that the natural shape of a woman's chest is not so elegant as we can make it, by the confinement of stays. The common effect of this practice is obstruction in the lungs, from their not having sufficient room to play, which, besides tainting the breath, cuts off numbers of young women in the very bloom of life. But nature has shown her resentment of this practice in a very striking manner, by rendering above half the women of fashion deformed, in some degree or other. Deformity is peculiar to the civilised part of mankind, and is almost always the work of our own hands. The superior strength,

* See a " Comparative View of the State and Faculties of Man with those of the animal World." London, 1772.

just proportion, and agility of savages, are entirely the effects of their hardy education, of their living mostly in the open air, and their limbs never having suffered any confinement."

But the deformity alluded to was not similar to that which is now prevalent: it consisted in a contracted form of the chest, accompanied by a degree of weakness that produced great distress. This condition of the chest, however afterwards aggravated, probably owed its origin to the custom of wrapping children in tight bandages almost as soon as they were born; for swaddling bands were formerly so common, that there are even directions given in books of surgery how they should be applied. The observations of the author quoted above are as forcible and just on this subject, as those which he has made on the use of stays: —"The evident tokens of delight which the little creature shows in recovering the free use of its limbs, and the strong reluctance it discovers to be again remitted to its bondage, one should think would strike conviction of the cruelty and absurdity of the custom into the most stupid of mankind."

Such arguments have had an influence, and children are now left in comparative freedom; but, notwithstanding the consequences of depriving any part of the body of the power of performing its natural functions, parents do not hesitate to swathe, and put into the most complete bondage, children of a more advanced age: for what are stays but bandages? However, as all the arguments that have been employed against the use of stays, and the proofs that have been given of their bad effects, will not prevent their being worn, our efforts must be directed towards rendering them as harmless as possible.

As even fashion does not require that a child should look otherwise than nature made it, there can be no necessity for putting a girl into stays before she is ten or twelve years of age. When stays *must* be put on, they should be loosely laced; for, the tighter they are, the more do they act as compressing bandages, which not only prevent the natural play of the muscles, and thus weaken them, but even waste and lessen their size. That such may be the effect of pressure, is often seen in the wasted leg of the mendicant, which, through tight bandaging alone, can be reduced to that condition which excites our commiseration.

If stays are put loosely on, and only worn occasionally; and if the girl takes sufficient active exercise, and rests in a proper manner when fatigued; there is little danger of the form suffering, even from strong stays. But (although, by this method, stays may be rendered almost harmless,) there will be some difficulty in pursuing it, as the girl will feel the occasional bondage very uncomfortable. The annoyance produced by it, is marked by the flushing of the face from impeded respiration, and by a stiff and constrained manner of walking. The remedy generally proposed is, that *she should wear the stays until she gets used to them.* This advice will probably be followed; and then it is likely that the bad effects, already described, will ensue.

But, on the other hand, it has still to be shown that stays may often be necessary, and that those generally used may, under certain circumstances, not be stiff enough.

Almost every step in the education of a young lady tends to make her artificial, at least as far as her body is concerned. Stays, tightly laced, are applied at an early age, and she is debarred from taking the exercises natural to youth; yet, notwithstanding the tendency of such a system to weaken all the muscles of the back, she is expected to be able to keep her spine as upright as if she had the strength of a porter. She may appear to sit erect, when she is, in fact, crooked: the cause of this is explained in the next chapter. But, to return to the question: a mistake seems to exist with regard to the effects produced by stays, which are not stiffened with bones. Mothers are led to believe that their children are in no danger of becoming distorted by wearing such stays; but they forget, or do not know, that the tight bandaging of the chest, when continued, is more injurious than the effect produced by stays which support the figure, even to such a degree as to obviate the necessity of the action of the muscles. It is true that a bandage, occasionally applied, gives support and strength; but, if constantly worn, it produces a wasting of the part. Proceeding on this view, it may be stated that, if tight stays *must* be worn, they should be made sufficiently stiff and strong to sustain the weight which the muscles, that have become deteriorated by want of action, are unable to support. If the stays are not made so, (the muscles, or natural means of support, being already weakened,) there is danger of certain ligaments of the spine yielding, and hence of the vertebræ falling out of their natural line, and thus producing curvature of the whole column. But, so prevalent is the persuasion that stays are injurious, and so little does the principle on which they are useful or hurtful seem to be understood, that the first thing generally recommended in a case of weakness, or yielding of the spine, is that the stays should be thrown aside; or, at least, that all the bones should be taken out. If a determined plan of practice, combining appropriate exercises with rest and proper support, is to be pursued, this may be all very well; but, when a girl is weak, to deprive her of her artificial supports, and to leave her at once to her own physical resources, seems to be acting in a manner very much at variance with the dictates of common sense. I have seen so many instances of the bad effects of this plan, that I cannot help expressing myself strongly.

Although a girl may be absolutely tortured when she begins to wear tight stays, she soon becomes so dependent on them as to feel very uncomfortable without them. If she should have a fit of illness, her dependence on this artificial support is more distinctly marked; for, when she recovers, and is expected to sit up, the muscles of her back are so feeble and relaxed, that she is not able, even during the short period of dressing, to keep herself erect, unless she binds the waist with a towel or handkerchief. A mother naturally takes alarm at this, especially when she recollects that, before the stays were worn, her daughter could not bear any thing tight round her, and was strong and active. She, however, hopes that the late illness is the sole cause of the weakness, and she waits in expectation that her daughter will become stronger; but the girl continues in the same dependent state, and probably complains of lassitude and a weary pain in the back. Her spine is now examined: perhaps a slight curvature is discovered, and pain when certain vertebræ are touched. She may now be condemned to lie for two years on the inclined plane: at all events, the bones are ordered to be taken out of her stays, under the idea that, as they have produced the distortion, all danger of the curve increasing will be removed by this measure.

But this only adds to the evil; for the girl has, from the weakness consequent on her illness, and the previous disuse of the muscles, fallen into such a condition as to require support. Although the supervention of distortion affords a good argument against the previous use of stays, they may nevertheless be necessary to a person in this state. Tight stays have been worn, the muscles have been weakened, distortion has taken place,—in short, the mischief is already done; and the question now is, not whether the stays produced the distortion, but what is to be done to counteract their previous effects. Surely we ought rather to add to the means of support for a time, than to take from them; and, therefore, when the spine yields, instead of throwing aside the stays, we should make them stronger,—and, if possible, in such a manner as to take off the weight of the shoulders and head from the lower part of the spine.

Although such stays will not cure a distorted spine, yet, if judiciously managed, they may tend to prevent the spine becoming more crooked, especially if they be combined with means of strengthening the muscles: indeed, common sense dictates, that, until we can restore the natural powers, by which the spine is enabled to support the upper part of the body, we should assist it by some artificial means.* But, although artificial supports are here recommended, they are to be considered as only assisting in the cure of distortion, and as the means of preserving what is gained in another way. In the preceding volumes, the question of the propriety of attempting to cure distortion by such machines, is discussed at some length. And, so far am I from swerving from the opinions expressed there, that I am more convinced than before of many of these machines being injurious in a variety of ways; and I would now object, even more than I formerly did, to that proposed by Mr. Chesher, for it is very lately that I became aware of its effect on the muscles of the fore-part of the neck. * * * Indeed, I have not known one instance, among a considerable number of patients who had worn this instrument, where it had not been in some degree hurtful. Some of these patients, notwithstanding the pain produced by the ulceration under the arms, wore it for a long time, in the hope of being eventually cured; but they were disappointed, for, although the ribs were modelled into a certain shape by the lateral pressure, the spine remained crooked. We cannot be surprised at this, as the muscles by which the spine is naturally supported are so weakened by the constant application of the machine, as to be rendered almost useless: indeed, in some instances, the spine and ribs have appeared as if covered only by skin, so much have the muscles been

---

* I lately received a very neat instrument, contrived for this purpose by M. Dupuytren, of Paris.

wasted. I do not know whether a cure has ever been effected where patients have worn the machine the time prescribed by the maker, (nine years!—that is, until the spine becomes fixed!) It has probably been seldom persevered in so long; for, in the course of two or three years, the patients become so weary of the constant pain and restraint, and the friends so tired of the frequent expensive repairs, that it is thrown aside; and then the mechanic's excuse is, that it has not had a fair trial. I have, in the preceding volumes, mentioned several of the evils attending the use of this instrument: were it necessary, I could offer several striking examples of its inefficacy and bad effects.

---

